# Strength Analysis of a Rib-Stiffened GLARE-Based Thin-Walled Structure

**DOI:** 10.3390/ma13132929

**Published:** 2020-06-30

**Authors:** Andrzej Kubit, Tomasz Trzepieciński, Bogdan Krasowski, Ján Slota, Emil Spišák

**Affiliations:** 1 Department of Manufacturing and Production Engineering, Rzeszow University of Technology, al. Powst. Warszawy 8, 35-959 Rzeszów, Poland; akubit@prz.edu.pl; 2 Department of Materials Forming and Processing, Rzeszow University of Technology, al. Powst. Warszawy 8, 35-959 Rzeszów, Poland; 3 Department of Mechanics and Machine Building, Carpatian State School in Krosno, ul. Żwirki i Wigury 9A, 38-400, Krosno, Poland; b_krasowski@wp.pl; 4 Institute of Technology and Material Engineering, Faculty of Mechanical Engineering, Technical University of Košice, Mäsiarska 74, 040 01 Košice, Slovakia; jan.slota@tuke.sk (J.S.); emil.spisak@tuke.sk (E.S.)

**Keywords:** aircraft fuselage skin, aluminium alloy, GLARE, fibre metal laminates, FML, mechanical engineering, thin-walled structures

## Abstract

This paper presents a new product, a glass laminate aluminium-reinforced epoxy (GLARE)-based thin-walled structure with a stiffener in the form of a longitudinal rib. The stiffening rib in an outer metallic layer of a GLARE-based panel was fabricated by the incremental sheet forming technique and Alclad 2024-T3 aluminium alloy sheets were used as adherends. The strength properties of the adhesive joint between the layers of the fibre metal laminates (FMLs) were determined in a uniaxial tensile test, peel drum test, tensile/shear test and short-beam three-point-bending test. Two variants of FMLs were considered, with an adhesive film and without an adhesive film between the adherends and the epoxy/glass prepreg. The FMLs were tested at three different temperatures that corresponded to those found under real aircraft operating conditions, i.e., −60 °C, room temperature and +80 °C. It was found that the temperatures do not affect the tensile strength and shear strength of the FMLs tested. However, there was a noticeable increase in the stiffness of samples stretched at reduced temperature. An additional adhesive film layer between the adherends and the glass/epoxy prepreg significantly improves the static peeling strength of the joint both at reduced and at elevated temperatures. A clear increase in the critical force at which buckling occurs has been clearly demonstrated in the uniaxial compression test of GLARE-based rib-stiffened panels. In the case of GLARE-based rib-stiffened panels, the critical force averaged 15,370 N, while for the non-embossed variant, it was 11,430 N, which translates into a 34.5% increase in critical force.

## 1. Introduction

Fibre metal laminates (FMLs) consist of alternating thin layers of metal sheets and fibre-reinforced prepreg epoxy. Glass, aramid fibres and carbon fibres are used as reinforcement. The FMLs with Kevlar fibres (aramid aluminium laminate—ARAL), carbon-reinforced aluminium laminate (CARAL) and glass fibres (glass laminate aluminium-reinforced epoxy) have been evaluated for potential applications in aircraft structures [1,2]. When compared to metallic materials, FMLs have a lower specific gravity, an ability to arrest the crack growth caused by cyclic loading and better damage tolerance, greater corrosion resistance, better impact strength, resistance to electric (atmospheric) discharges and an inherent high burn-through resistance [3,4,5].

The most important advantage of FML hybrid layer composites is their high fatigue strength, resulting from slow crack propagation. The mechanism of the fatigue destruction of such materials is different from that of metals and other fibre-reinforced polymer composites, and is characterised by multi-fracture modes such as delamination, matrix cracking, fibre fracture, fibre-matrix debonding and fibre/matrix interfacial shear failure [1]. The identification and detection of these defects in the glass laminate aluminium-reinforced epoxy (GLARE) composites is an important task for safety assurance in the aircraft industry [5,6,7].

High-performance FMLs are attractive for lightweight, fatigue-critical structural applications. The first commercial application of GLARE laminate was in the fuselage skin of the Airbus A380, which consists of about 350 m^2^ of GLARE [8]. GLARE may also be used in the leading edge of the wings and tails of the Airbus A380 [1]. GLARE has also been selected for the Boeing 777 impact-resistant bulk cargo floor [4]. The Boeing 787 Dreamliner was the first commercial airplane to be constructed from 50% of different types of composite materials [9]. A number of advanced investigations preceding the introduction of FMLs into the construction of aircraft have demonstrated the significant advantages of this type of hybrid material. It has been confirmed [1,10,11,12,13] that FML composites, due to their layered structure, have the property of limiting the propagation of fatigue cracks. Compared to glass fibre-reinforced polymers (GFRPs) and carbon fibre-reinforced polymers (CFRPs), FMLs are characterised by high impact strength [7,14,15]. At the same time, FML composites are characterised by low density [7]. An important advantage of this type of laminate is the possibility of joining large thin-walled structures by means of adhesive bonding, which eliminates the mechanical riveting typical of metallic structures in which it is necessary to make holes [12,16,17].

Glare laminates are manufactured by bonding together fibre composite prepreg and metal sheets using a press, or more often, an autoclave. An autoclave process greatly increases the cost of manufacture of high strength FML-based structures, which limits their widespread use. Although a number of alternative out-of-autoclave methods that can allow one to replace the autoclave curing process—such as induction heating [18], microwave radiation [19] or resistance heating [20]—have been investigated for curing carbon fibre-reinforced thermoset composites, it is difficult to achieve the desired strength properties in the structures thus produced. An increasingly large quantity of voids resulting from the lower pressure applied during curing, which reach content values of up to 20% [9], limits the application of non-autoclave manufacturing methods.

Despite the current technological limitations, it can be assumed that GLARE composites will acquire an important role in the construction of thin-walled aircraft structures in the future [21,22,23] due to their very attractive properties. The global trends forcing ecological solutions for means of transport resulting from significant restrictions on the emissions of CO_2_ and nitrogen compounds will also be a commercial catalyst for the development and dissemination of lightweight hybrid composites. Typical aircraft skins made of GLARE composites have a classic structure, where the metallic stringers and frames are joined to a skin made of multilayered composite, usually by adhesive methods.

**Figure 1:**
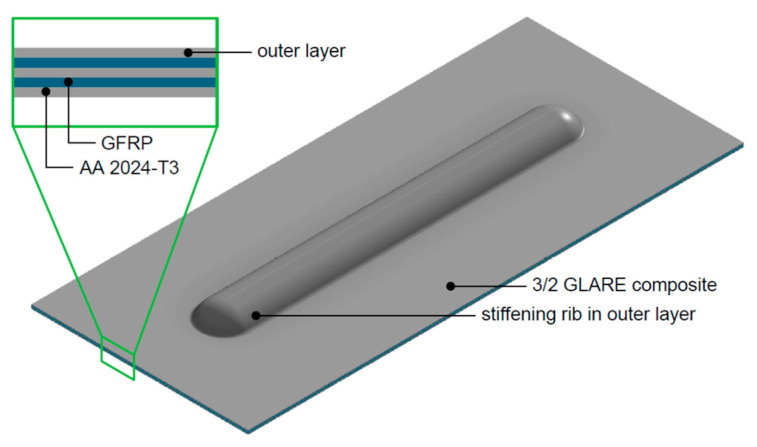
Concept of a glass laminate aluminium-reinforced epoxy (GLARE)-based (3/2 lay-up) structure with a stiffening rib on the outer metallic sheet.

The hybrid FMLs may also be used in the construction of less critical elements of the aircraft structure, such as barriers, floor elements and other elements that do not affect flight safety. However, these structural elements are extremely important for weight reduction purposes. Hence, any construction solution improving strength and stiffness without increasing weight is important, worth considering and to be welcomed. It is for this reason that we present a novel solution in this paper consisting of a GLARE-based thin-walled structure with a stiffener in the form of a longitudinal rib (Figure 1) fabricated by incremental sheet forming (ISF). In ISF, the degree of sheet deformation is higher than in conventional die forming. The GLARE composite consists of Alclad 2024-T3 aluminium alloy sheets, commonly used in the aerospace sector, and a GFRP (3/2 lay-up). The strength properties of the adhesive joint between the layers of GLARE composite were determined in a uniaxial tensile test, tensile/shear test, peel drum test and short-beam three-point-bending test. The effect of the stiffening rib on the strength of the panel was verified in a compression test.

## 2. Materials and Methods

### 2.1. Materials

Alclad 2024-T3 aluminium alloy sheets with thicknesses of *t* = 0.4 and *t* = 2 mm, which are widely used in the aircraft industry to fabricate fuselage skins, were used as the adherend. The mechanical properties of the aluminium alloy sheets used were determined in a uniaxial tensile test using a Z100 (Zwick/Roell, Ulm, Germany) universal testing machine at 24 °C according to the ISO 6892-1 standard [24]. The average values of the basic mechanical parameters listed in Table 1 were determined based on five measurements.

Prior to bonding, the surfaces of the adherend were anodised according to the following procedure. The samples were abraded with sandpaper (grade 320), rinsed with water, degreased in a solution of sodium hydroxide (100 g·dm^−3^) with water for 1 min, rinsed with deionised water, and pickled in a solution of nitric acid (400 g·dm^−3^) with water for 1 min at 25 °C. Next, the specimens were anodised in an aqueous solution of sulphuric acid (300 g∙dm^−3^) at 15 °C at a current density of 1 A·dm^−2^. The anodising time was equal to 25 min and 10 min in the case of an adherend thickness of 2 and 0.4 mm, respectively. After the anodisation process, the coatings obtained were rinsed with deionised water and dried in air. The aluminium alloy surfaces were primed with structural adhesive primer EC-3924B by 3M™ with the following characteristics: base, synthetic resin; flash point, 14.4 °C; density, 0.887 kg·dm^−3^; solids content, 6 ± 1.0%.

**Figure 2:**
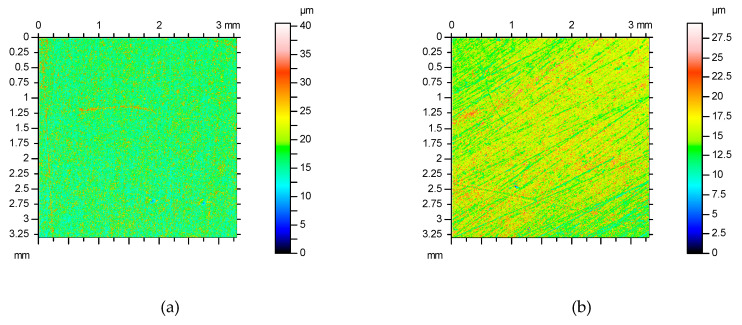
2D profiles of measured sheets with thicknesses of (**a**) 2 mm and (**b**) 0.4 mm.

The topography of the 2024-T3 aluminium alloy sheets was tested with a Talysurf CCI Lite 3D optical measurement system. The basic surface roughness parameters—i.e., the 10-point peak-valley surface roughness *Sz*, the highest peak of the surface *Sp,* the texture aspect ratio of the surface *Str*, the maximum pit depth *Sv*, the root mean square roughness parameter *Sq*, the surface kurtosis *Sku*, the surface skewness *Ssk* and the roughness average *Sa*—were determined before the application of the primer. The basic surface roughness parameters determined on the basis of five profiles with an area of 3.3 × 3.3 mm are listed in Table 2. The surface roughness topographies of the original sheets are shown in Figure 2.

**Figure 3:**
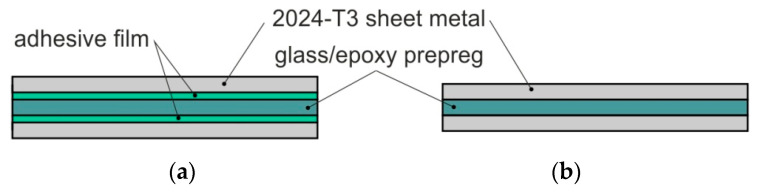
Stacking configurations of the fibre metal laminate (FML) (2/1 lay-up) variants considered: (**a**) with adhesive film; (**b**) without adhesive film.

Two variants of FMLs consisting of a five- and three-ply lay-up were fabricated. According to [25], “*the application of the adhesive film as an additional binding agent caused an increase in laminate elasticity*”, which results in a significant increase in the peel strength of the laminate. Thus, in this paper, two variants of FMLs were considered, with adhesive film and without adhesive film between the adherends and glass/epoxy prepreg. In the first configuration of the laminate (Figure 3b), the FML was prepared without adhesive film between the matrix and adherends. In the second type of FML (Figure 3a), the 3M Scotch-Weld^TM^ AF-163-2K (3M^TM^, Saint Paul, MI, USA) thermosetting modified epoxy adhesive film was used as an intermediate layer between the adherends and glass/epoxy woven HEXPLY-916G (Hexcel Corporation, Stamford, Connecticut, USA) prepreg. 

**Figure 4:**
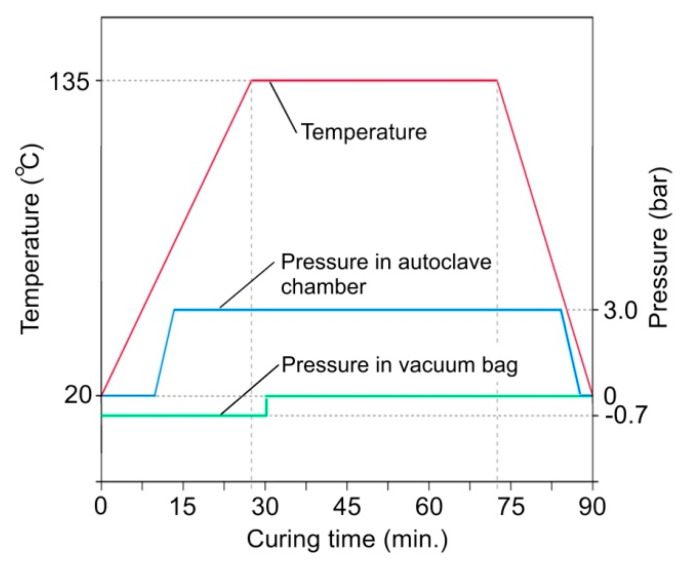
Autoclave cure cycle.

The autoclave cycle for laminate fabrication is presented in Figure 4. The FML panels are cured at a temperature of 135 °C for approximately 45 min. The heating and cooling speeds were equal to 2 °C·min^−1^ and 3 °C·min^−1^, respectively. The vacuum bag and autoclave pressures were maintained at −0.7 bar and 3 bar, respectively.

The specimens used for the strength tests were cut using a water jet (WJ) technique. The WJ cutting was carried out at a speed of 250 mm·min^−1^, with an abrasive mass flow rate of 300 g·min^−1^ and a water pressure of 350 MPa.

### 2.2. Uniaxial Tensile Test

The guarantee of quality that ensures that the layered composites have the required strength properties is a high-strength interlayer adhesive connection. Because it is proposed to use the FLMs with an additional stiffening rib investigated in this study in applications for the construction of thin-walled aircraft structures, a number of tests were carried out under different temperature conditions that corresponded to the real operating conditions of aircraft. It has been assumed that the extreme temperatures at which the aircraft structure can operate are −60 °C when flying at an altitude of 12,000 m and an upper limit of +80 °C when standing on the tarmac in the hottest regions of the world [26].

**Figure 5:**
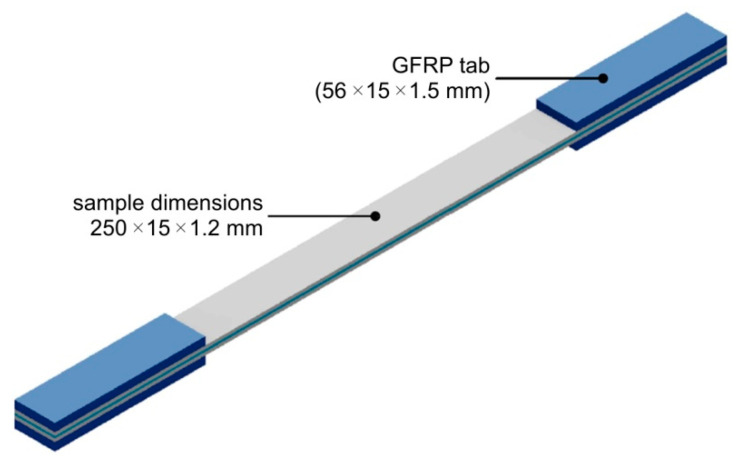
Dimensions of the FML in a variant of a 2/1 lay-up used in uniaxial tensile tests (GFRP—Glass Fibre Reinforces Polymer).

The experimental procedure for the uniaxial tensile testing of specimens in a variant of a 2/1 lay-up was based on the ASTM standard D3039/D3039M-17 [27]. The dimensions of the specimens are presented in Figure 5. Testing was carried out using a Z100 universal testing machine (Zwick/Roell, Ulm, Germany) equipped with a temperature chamber and with a maximum capacity of 100 kN. Tensile tests were performed at temperatures of –60 °C and +80 °C. A compact cryostat (cryogenic agent: liquid nitrogen, LN_2_) with mechanical testing appliances was used to ensure a temperature of −60 °C during tests. Prior to testing, the samples were held in the cryostat at the specified temperature for 15 min to achieve a uniform temperature throughout the sample. The samples for testing at elevated temperature were heated up to +80 °C and stored at such a temperature for 15 min. The temperature in the temperature chamber was controlled by the testXpert® software (Zwick/Roell, Ulm, Germany). Five specimens were tested to evaluate the average tensile strength of the FML. The testing speed was 5 mm·min^−1^.

### 2.3. Tensile/Shear and Drum Peel Tests

**Figure 6:**
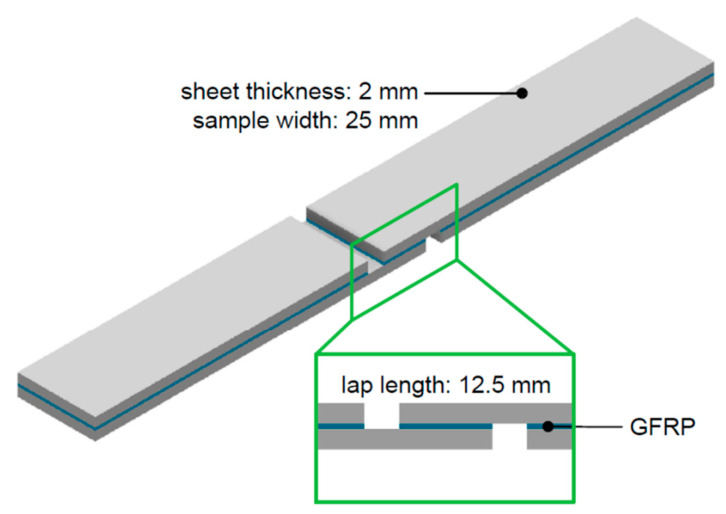
Sample for testing the interlaminar strength of the FML composite in the static tensile/shear test.

In the next stage of the research, the strength of the interlayer adhesive bond in the FMLs being investigated was tested with a static tensile/shear test. The shape and dimensions of the specimen are shown in Figure 6. To ensure a suitable rigidity of the specimen, 2 mm-thick adherends were used to allow the tests to be carried out under pure shear conditions. Three specimens were tested to evaluate the average tensile/shear strength of the laminate.

**Figure 7:**
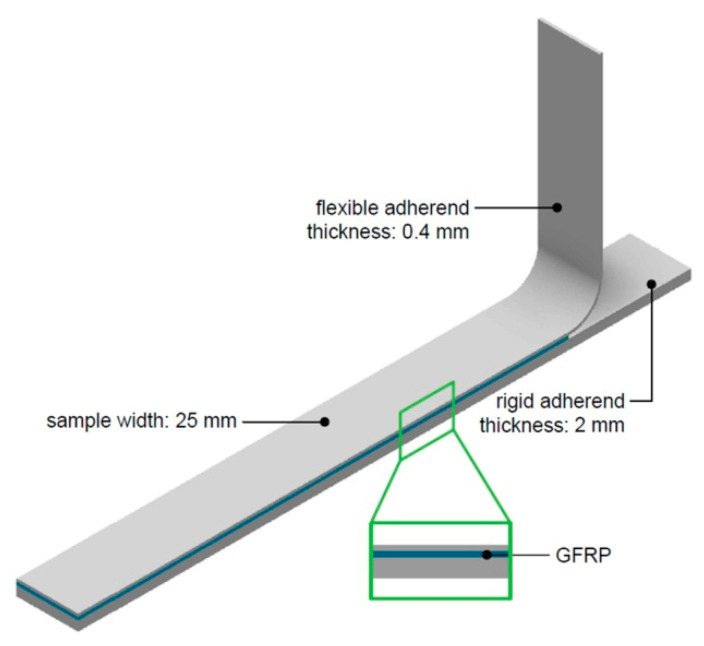
Sample for testing the interlaminar strength of the FML composite in the drum peel test.

According to this principle, samples for a drum peel test (Figure 7) were also prepared using a 0.4 mm-thick sheet as a flexible adherend and a 2 mm-thick sheet as a rigid adherend. Three specimens were tested to evaluate the average peel strength of specimens.

### 2.4. Short-Beam *Three-Point-Bending* Test

**Figure 8:**
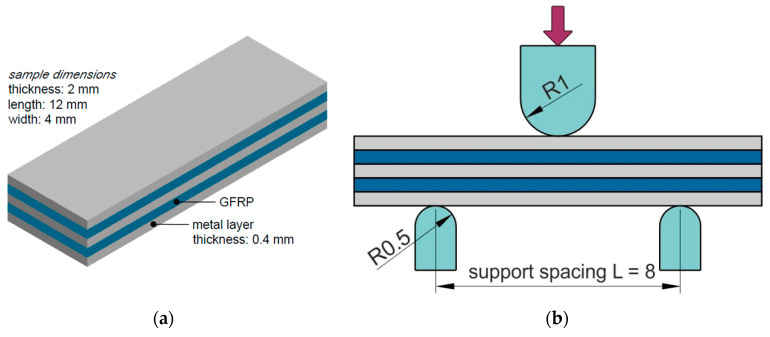
(**a**) Shape and dimensions of the specimen and (**b**) schematic diagram of the short-beam three-point-bending test (dimensions in mm).

The 7000 and 2000 series aluminium alloys used in aviation, which are produced by including additives in the alloys that ensure that they have adequate mechanical parameters, are susceptible to different forms of atmospheric corrosion such as intergranular corrosion, pitting corrosion and even exfoliation corrosion [28]. An important advantage of FML composites is that they provide protection of the internal metallic layers against corrosion by joining their entire surfaces with epoxy resin. Hence, in typical GLARE composites, the sheets are anodised, which assures sufficient protection against corrosion. However, in the configuration analysed in this paper, the GLARE-based rib-stiffened panel is not coated with epoxy resin in the region of the rib. Thus, in this area, Alclad 2024-T3 aluminium alloy is used. In order to determine the effect of the Alclad coating on the mechanism of destruction of the interlaminar joints in the FML, a short-beam three-point-bending test was performed. The ASTM standard D2344 [29] recommends that the span-to-thickness ratio of a short-beam specimen be 4 to 5 and that the test yields apparent interlaminar shear strength. A relatively small span-to-thickness ratio as recommended by ASTM D2344 may cause local buckling due to possible local waviness in the GLARE layers and lower shear strength [30]. Samples were used in the 3/2 lay-up configuration with the dimensions shown in Figure 8a, while in the three-point bending test, a support spacing of 8 mm (Figure 8b) was used. Three specimens were tested to evaluate the load–deflection curves of specimens.

### 2.5. Fabrication of GLARE-Based Rib-Stiffened Panels

**Figure 9:**
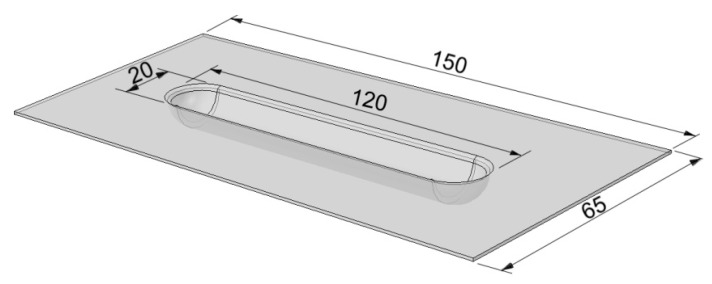
Shape and dimensions of the rib-stiffened panel.

The trials in relation to the formation of longitudinal U-shaped stiffening ribs (Figure 9) by ISF were conducted on the CNC HAAS TM1P 3-axis milling machine using a special fixture mounted on the bed of the machine tool. To reduce the contact forces on the tool tip–sheet metal interface, a fully synthetic 75W85 oil (Castrol Ltd., UK) was applied. The basic properties of the oil used are a viscosity of 74.0 mm^2^·s (at 40 °C), density of 874 kg·m^−3^ (at 15 °C) and freezing point of −45 °C. A sheet is placed in the tooling and is clamped at the edges. The tool then moves, tracing the required shape in the space under CNC control, so that the part is obtained as the result of accumulated, localised, plastic deformations. 

The tool had a rounded tip with a radius R of 3.5 mm and was made of high-speed steel. The tool was clamped in the head of the machine using the “ER” collet system, which allowed the tools to be mounted with a cylindrical shank. The parameters of the forming process are as follows: feed rate *f* = 800 mm·min^−1^, tool rotational speed *n* = 96 rpm and vertical pitch *a_p_* = 0.5 mm. The part strategy is that the tool moves downwards to the final position along a continuous path with a linear vertical pitch. The profile of the tool-path trajectory for the desired geometry was generated using the EdgeCAM software. 

### 2.6. Buckling Test

**Figure 10:**
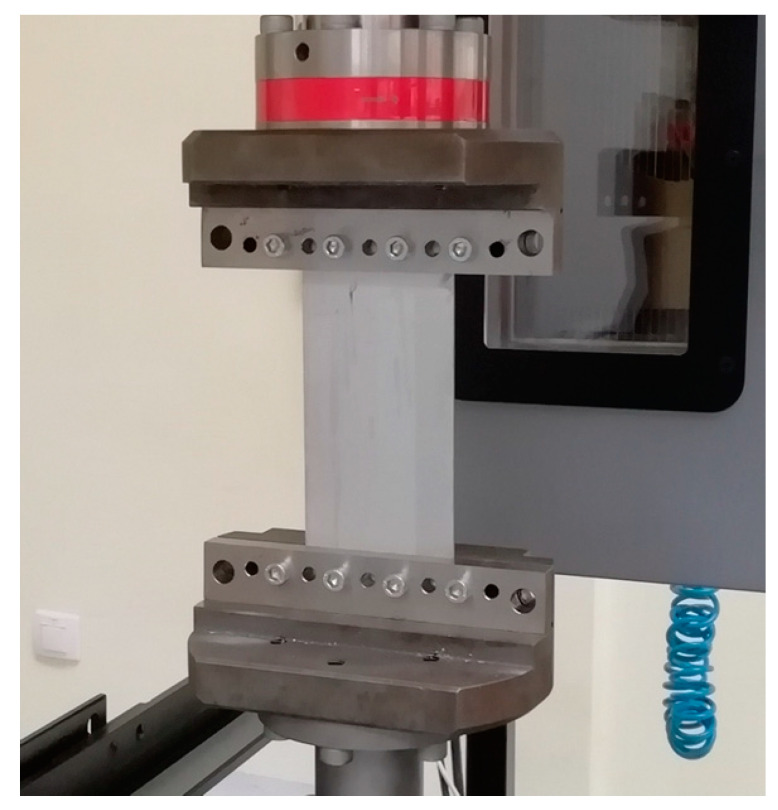
Equipment for the buckling experiment.

When investigating the structures made of these FMLs for their practical use in aircraft and civil engineering, the experiments often produced a variety of actual buckling behaviours [31]. Conducting buckling experiments is a powerful approach for researching types of composite materials [32,33]. Experimental tests on the buckling of FML profiles were performed using a universal strength testing machine manufactured by Instron (Norwood, MA, USA) with a maximum load range of 100 kN in uniaxial compression mode. In order to be able to carry out the buckling test, the tensile machine is equipped with an apparatus that can meet the required boundary conditions (Figure 10). Two opposite edges of an FML panel were selected as free, and the other two opposite edges were screwed in an apparatus for the boundary conditions. The buckling tests were performed at a test speed of 0.5 mm·min^−1^. Two variants of the FML (3/2 lay-up) were tested, those with and those without stiffening ribs. The tests were continued until the samples were destroyed.

### 2.7. Microstructural Analysis

The morphologies of the fracture surfaces of the specimens were examined using the (SEM) S-3400 scanning electron microscope (Hitachi, Chiyoda, Japan). The chemical composition of the intermetallics was analysed by energy-dispersive spectroscopy (EDS) with a spectrometer attached to the scanning electron microscope.

## 3. Results and Discussion

### 3.1. Uniaxial Tensile Test

**Figure 11:**
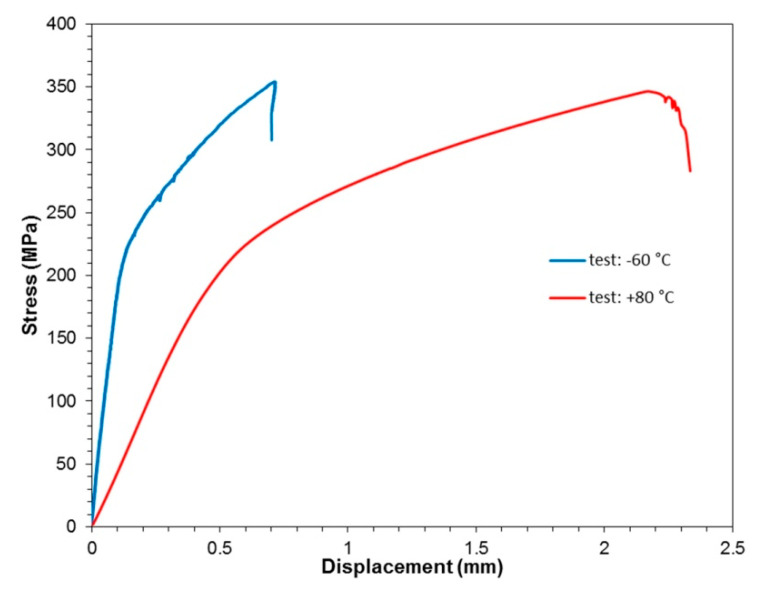
Tensile curves from the static tests at various temperatures.

The tensile curves for tests carried out at the three temperatures evaluated are shown in Figure 11. In the static tensile tests of the 2/1 lay-up FML, it was shown that the composite has similar mechanical properties at a reduced temperature of −60 °C to those found at room temperature. In the range of elastic strains, up to the yield point, the samples exhibit an almost identical stretch course. In the area of plastic deformations, on the other hand, an increase in the stiffness is noticeable in the samples stretched at reduced temperature. A significant reduction in FML stiffness in the elastic–plastic range occurs at elevated temperature. It was found that in the temperature range considered, there are no significant changes in tensile strength, which is equal to 354.7 ± 4.61 MPa for samples tested at −60 °C and 347.9 ± 7.03 MPa for the tests conducted at an elevated temperature.

The dominant mechanism of destruction in the metal layers of the laminate is sheet cracking preceded by the processes of plastic deformation. The degradation of FML layers can take a variety of forms depending on the type of layer components and loading conditions. In the case of FMLs based on thermosetting polymer resins reinforced with the most popular types of filaments, i.e., glass and carbon fibres, the destructive processes are related to the load direction relative to the orientation of the reinforcing fibres.

**Figure 12:**
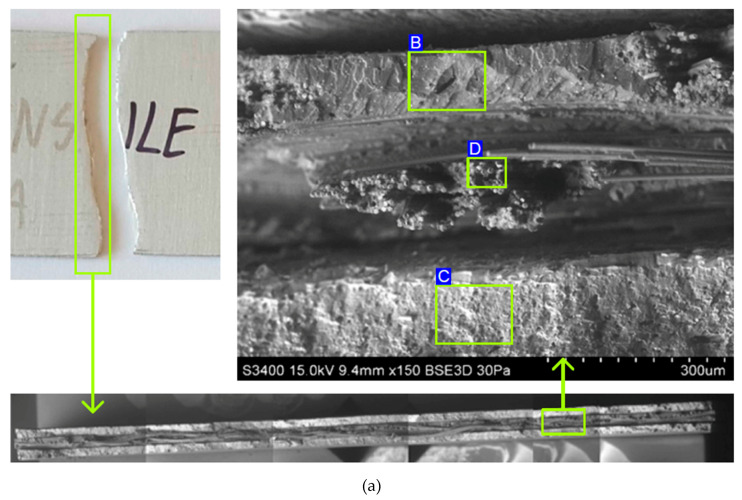

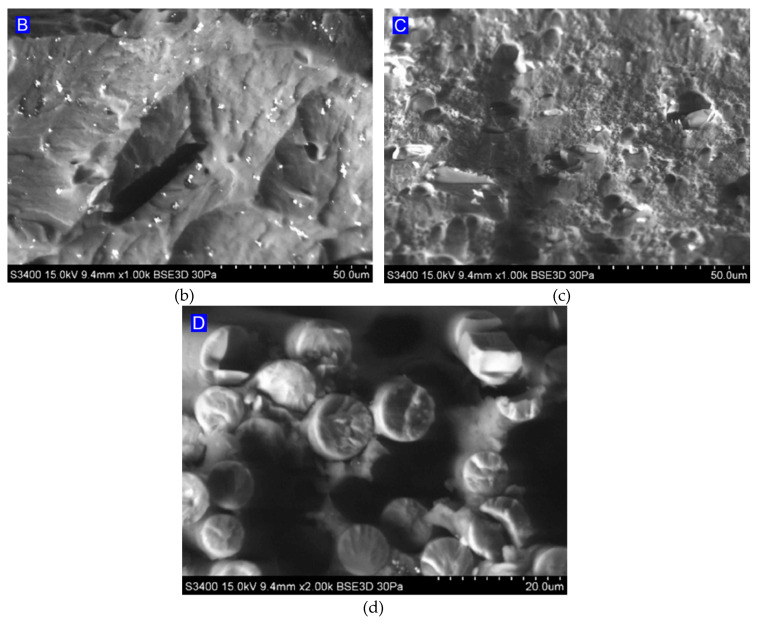
SEM micrographs of the fracture surfaces of samples subjected to the static tensile test at RT: (**a**) view of all layers, (**b**) fracture epoxy resin, (**c**) fracture of sheet metal, and (**d**) broken glass fibres.

Figure 12 shows SEM fractography of the fracture of a specimen tested at (RT). From the pictures of Figure 12a, it can be seen that before the final destruction of the sample by stretching, there was a delamination between the metallic layer and the GFRP. This was the result of shearing between layers with significantly different properties. Although the course of the stretching curves for RT and −60°C did not show any clear disturbances of the increase in the tensile force before the moment that the sample broke, the possibility of microcracks forming in the material or on the interfaces could be revealed. With the increase in temperature, the plastic mechanical properties of the constituent materials improved. It was found that a rise in temperature does not weaken the intermolecular forces between the aluminium alloy sheets and the matrix, resulting in similar tensile strength.

### 3.2. Tensile/Shear Test

**Figure 13:**
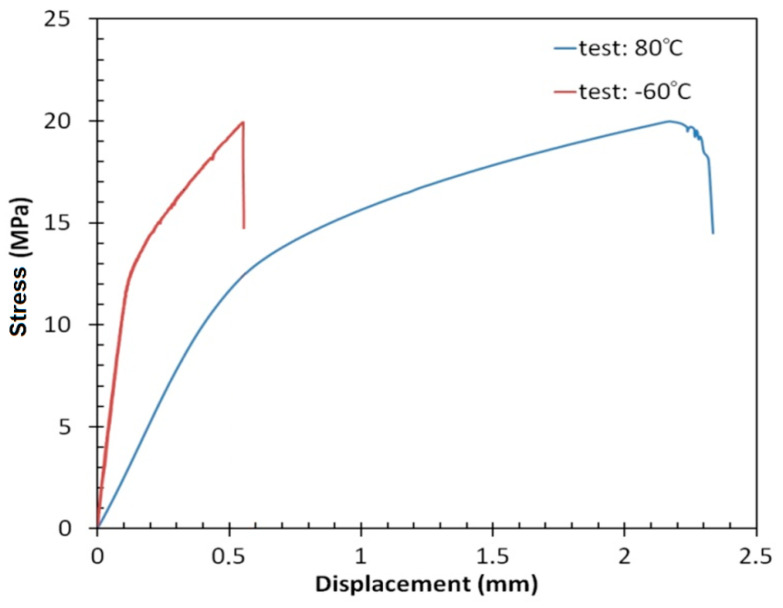
Stress–displacement curves after tensile/shear tests.

As a result of the static shear strength tests of the adhesive connection between the layers of FMLs, it was shown that in the elastic range, the connection behaves differently depending on the temperatures considered (Figure 13). Samples tested at a temperature of 80 °C show greater elasticity than samples tested at reduced temperatures. Therefore, it can be concluded that during the operation of such a structure, cyclic temperature variability has a significant effect on the mechanical and stiffness properties of the structure. After exceeding the yield point, a clear difference in the course of the shear test curves was observed. At −60 °C, a very wide area of plastic deformation of the adhesive joint was observed, which was finally destroyed at an average stress value of 19.95 ± 2.95 MPa. Samples subjected to testing at elevated temperature were characterised by a curve with a small plastic area, and the samples were rapidly damaged at significantly lower values of shear stress; the average shear strength was 19.90 ± 2.06 MPa.

**Figure 14:**
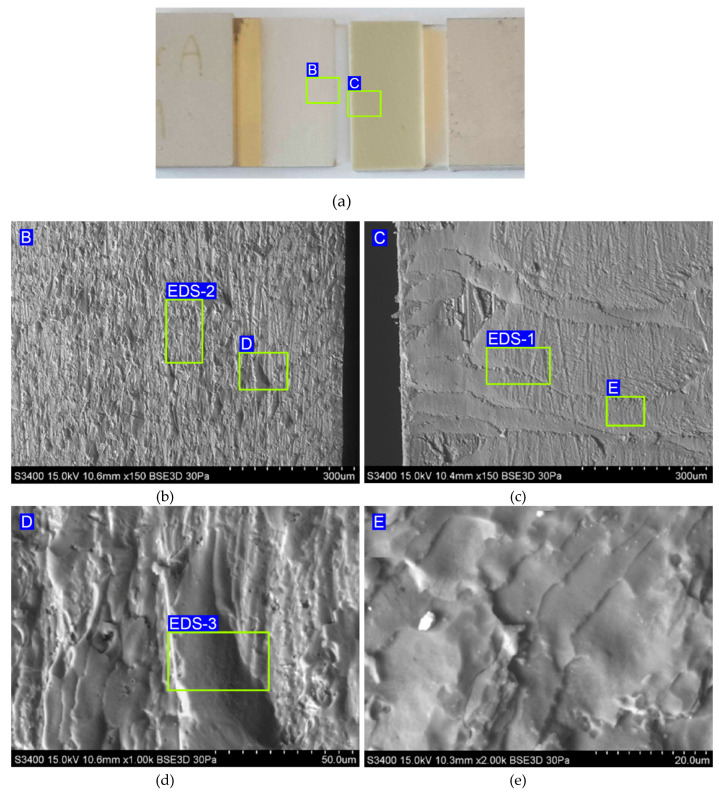
(**a**) Macrograph of the specimen tested at −60 °C with both sides of the failure and (**b**–**e**) magnifications of the surface of the fracture.

The analysis of the fracture surface of specimens tested at −60 °C, based on imaging on a macroscopic scale (Figure 14a), led to the conclusion that there was adhesive failure. A similar observation is reported for the specimen tested at 80 °C. However, when looking at the SEM images on a micro scale, it can be seen that epoxy resin residues are visible on the sheet surface (Figure 14b), which is not compatible with the original hypothesis of adhesive failure. Based only on SEM micrographs, however, it cannot be determined whether there was failure between the primer and the metal sheet, or between the primer and the epoxy resin, because, before curing in the autoclave, the sheets were originally coated with a primer which formed a thin, transparent layer. Thus, it was decided to assess the mechanism of failure based on the chemical composition. EDS analysis was performed for the surfaces with matrix (Figure 14c,e) to determine the chemical composition of the epoxy resin, which can be clearly identified. 

**Figure 15:**
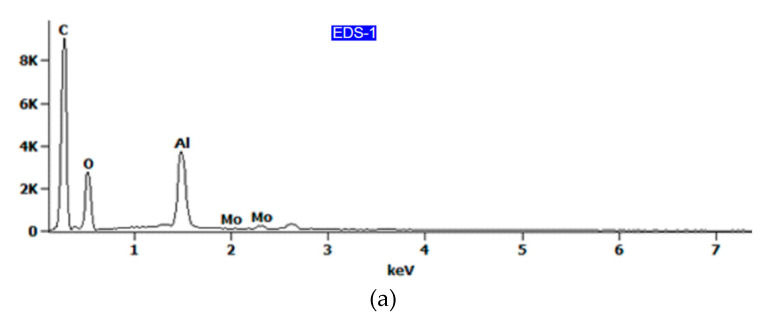

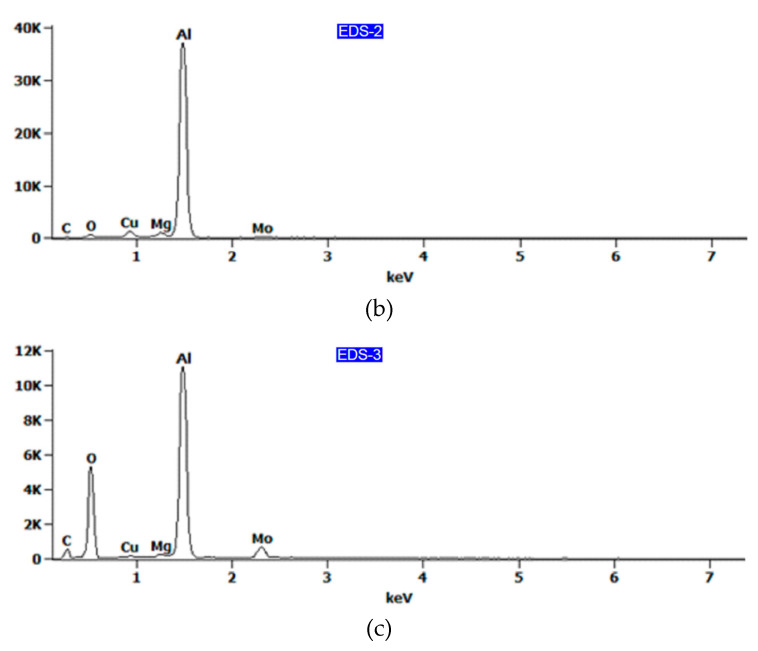
EDS spectra for the fracture surface of (**a**) epoxy resin and (**b**,**c**) aluminium alloy sheet.

The EDS spectra are shown in Figure 15, while the chemical composition is listed in Table 3. It has been shown that epoxy resin can be identified here as carbon–oxygen compounds, which coincides with the chemical composition of the resin. At the area-designated EDS-2 (Figure 14b) and EDS-3 (Figure 14d), located at the fracture surface of the aluminium alloy sheet, the results of the analysis of the chemical composition revealed that the aluminium was combined with copper and magnesium (Figure 15b,c), which corresponds to the 2024-T3 aluminium alloy used. Therefore, it can be concluded that the connection between the metal sheet and the primer was damaged, which is demonstrated on the fractured surface by residuals of resin (area EDS-1 in Figure 14c). EDS analysis of the surface of the aluminium alloy sheet (area EDS-3 in Figure 14d) clearly demonstrated the combined cohesive failure in the resin and adhesive failure in the primer.

In the case of rough surfaces and for the elements such as С, O, and N, errors in EDS analysis (Figure 15a) can be high. For example, it is very hard to determine the oxygen concentration in thin films on the surface of aluminium as there is always an oxide layer on the aluminium, and it is impossible to separate the concentrations of oxygen in the adhesive film from those on the aluminium surface. However, the results presented could be helpful in the qualitative assessment of the chemical composition of the fracture surfaces.

### 3.3. Drum Peel Test

**Figure 16:**
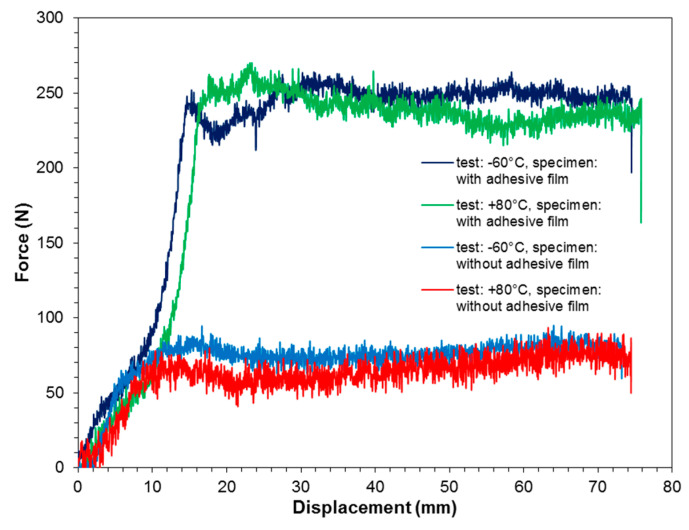
Load–displacement graph measured during drum peel tests.

Based on the results of drum peel peeling tests, it was confirmed that the use of an additional adhesive film layer significantly improved the static peel strength of the joint both at reduced and at elevated temperature (Figure 16). For the variant with adhesive film tested at reduced temperature, an average peel strength of 9.93 ± 1.07 N·mm^−1^ was obtained, while at elevated temperature, the strength slightly decreased to an average of 9.60 ± 1.99 N·mm^−1^. Thus, no significant effect of temperature on peel strength was demonstrated in the FML variant with adhesive film. However, a typical reduction in joint stiffness was observed at elevated temperatures.

The situation is similar for the joint produced directly by the prepreg (without adhesive film). A decrease in the stiffness of the joint at elevated temperature was noticed, while the reduction in the peel strength of the adhesion joint was more significant. At an elevated temperature, an average peel strength of 2.68 ± 0.25 N·mm^−1^ was observed, while at −60 °C, the average strength value was 3.04 ± 0.19 N·mm^−1^.

**Figure 17:**
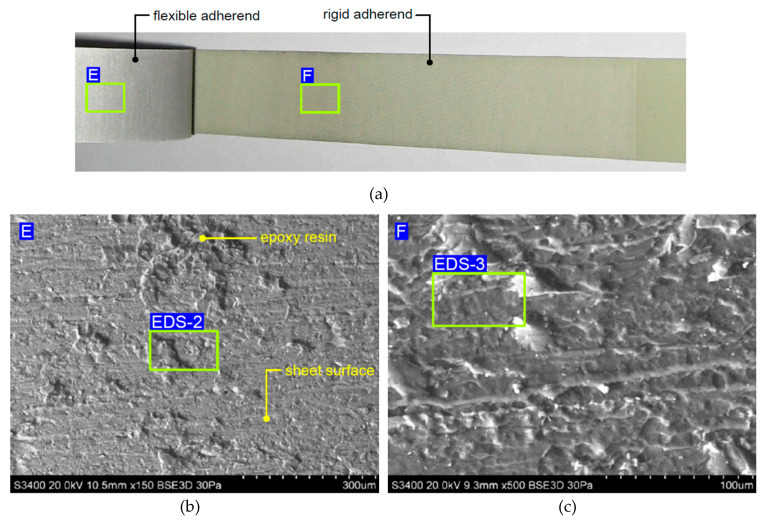
(**a**) View of the specimen without adhesive film, and SEM micrographs of fractured surfaces on the (**b**) flexible adherend and (**c**) rigid adherend.

Figure 17 presents view of the specimen without adhesive film (Figure 17a), and SEM micrographs of fractured surfaces on the flexible adherend (Figure 17b) and rigid adherend (Figure 17c). The joint produced by the epoxy resin, which is the impregnant of the prepreg, is characterised by significantly less adhesion, since only a small amount of the epoxy resin remains on the surface of the flexible adherend (area EDS-2 in Figure 17b).

**Figure 18:**
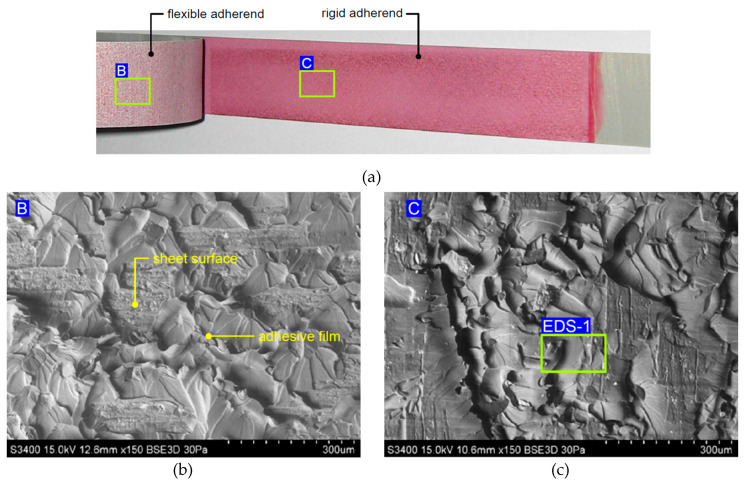
(**a**) View of the specimen with adhesive film, and SEM micrographs of fractured surfaces on the (**b**) flexible adherend and (**c**) rigid adherend.

**Figure 19:**
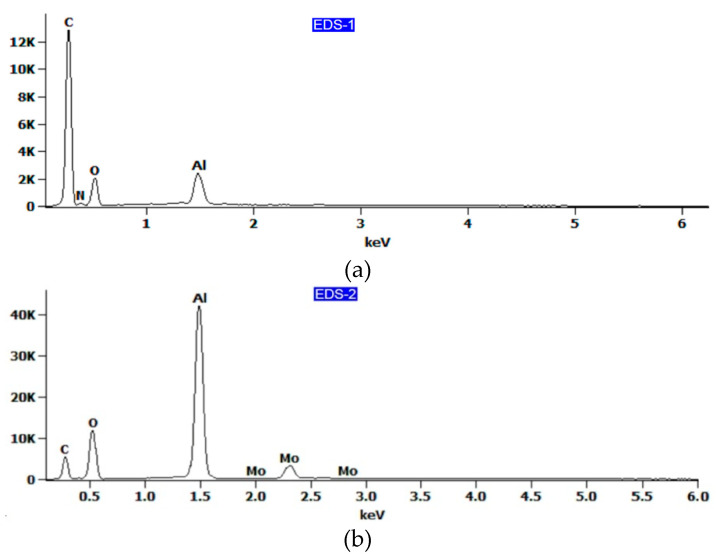

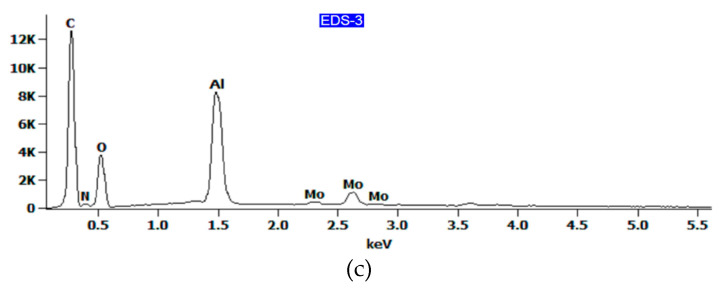
EDS spectra for the fractured surfaces of the (**a**) flexible adherend in a variant with adhesive film and of the (**b**) flexible and (**c)** rigid adherends in a variant of FML without adhesive film.

It was observed that in the variant of FML with additional adhesive film (Figure 18a), there was a combined adhesive and cohesive mechanism of fracture. Samples with an additional adhesive film exhibited stronger adhesion than FMLs without this film because the surface of the sheet was slightly exposed on the surface of the flexible adherend dominated by hardened epoxy resin (Figure 18b). The results of the EDS analysis (Table 4) revealed that the chemical composition of the adhesive film (area EDS-1 in Figure 18c) mainly consisted of carbon and nitrogen (Figure 19a, Table 4).

The main elements existing on the surface of the flexible adherend on an FML variant without an additional adhesive layer (Figure 17a) are oxygen and aluminium (Figure 19b, Table 3), which can be interpreted as the oxide layer resulting from the anodisation process. The main elements existing on the surface of the rigid adherend on an FML variant without an additional adhesive layer (area EDS-3 in Figure 17c) are carbon and aluminium (Figure 19c).

**Figure 20:**
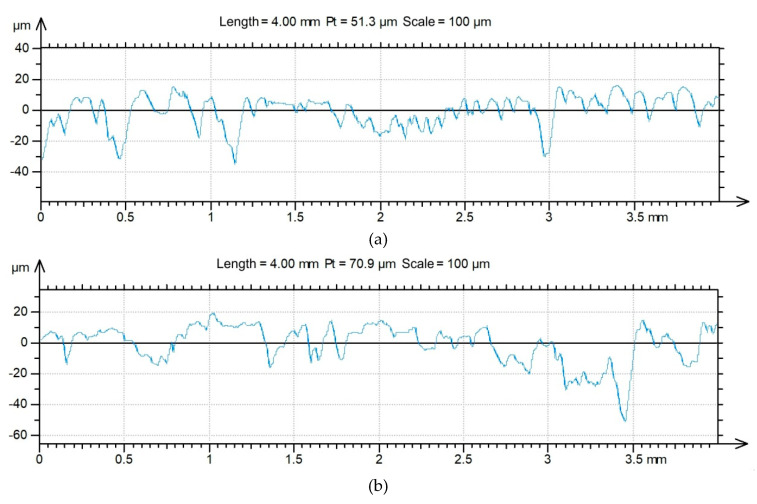
2D profile of the fractured surface of the rigid adherend in a variant of FML with adhesive film, measured for the specimen along the (**a**) longitudinal and (**b**) transverse directions.

**Figure 21:**
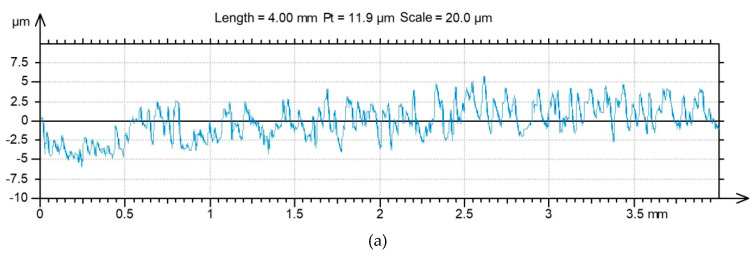

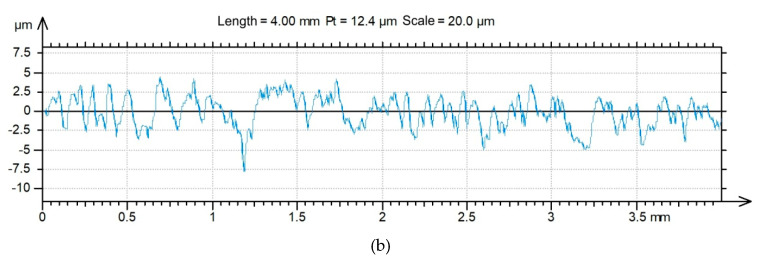
2D profile of the fractured surface of the rigid adherend in a variant of FML without adhesive film measured for the specimen along the (**a**) longitudinal and (**b**) transverse directions.

Surface roughness profiles of the fractured surfaces on the rigid adherend are shown in Figure 20 and Figure 21 for specimens with and without adhesive films, respectively. Moreover, the values of the surface roughness parameters are shown in Table 5.

### 3.4. Short-Beam *Three-Point-Bending* Test

**Figure 22:**
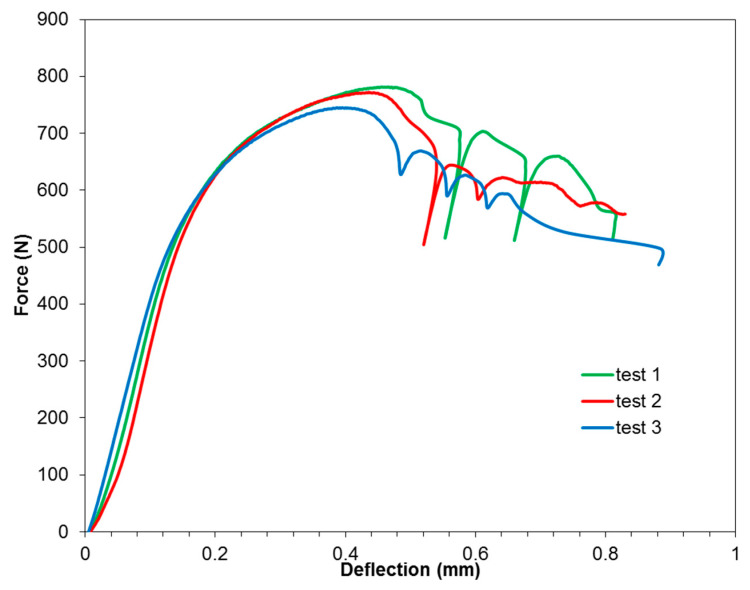
The load–deflection curves of 3/2 lay-up specimens.

**Figure 23:**
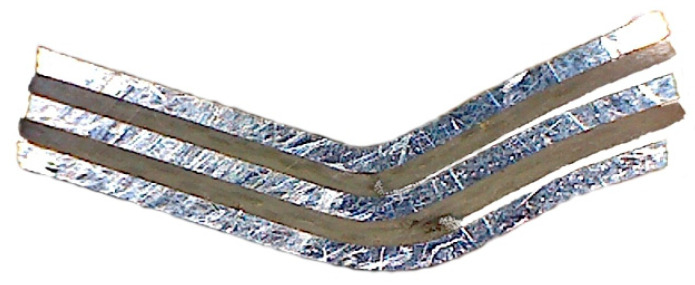
Failure mode after short-beam three-point-bending test with a span-to-thickness ratio of 4.

The short-beam three-point-bending test was carried out in order to determine the effect of the Alclad layer on the adhesive connection between the composite layers at room temperature. Typical GLARE composites are fabricated from uncladed sheets, while in our investigations, due to the unprotected inner surface of the stiffening rib, it was decided to use an Alclad sheet with the additional corrosion protection of the GLARE-based stiffened panel. Figure 22 shows the load–deflection curves from a short-beam three-point-bending test. After maximum load, each curve displays an irregular fluctuation, demonstrating that multiple failures took place, which is consistent with the observations of Liu et al. [30]. Shear-dominant failure was observed for the test specimens (Figure 23). This could be explained by the lower shear strength of the FML leading to local buckling so that the specimens deformed easily with the span-to-thickness ratio of 4 that was used. 

SEM observations of the fracture surfaces revealed that the Alclad layer was folded between the areas of contact of the specimen with the loading noses. In the area where pressure was applied, wrinkling and cracking of the Alclad was observed when the largest deformation of the specimen took place, which consequently led to a loss of connection integrity. The phenomena mentioned have a significant impact on the mechanism of FML failure, but it should be noted that they appear within the range of the plastic deformation of the sample. Thus, it can be concluded that during the normal operation of FMLs in the range of elastic strains, the Alclad has no significant effect on the quality of the joint. It may be concluded that the connection between the Alclad 2024-T3 sheets and the glass/epoxy prepreg is created between the epoxy resin and the aluminium oxide layer formed during the anodisation process.

### 3.5. Buckling Test

The guidelines for assuring a proper adhesive connection between the individual layers of the conceptual GLARE-based thin-walled structure with a stiffening rib were determined on the basis of the results noted above. It has been proven that during normal operation, when the structural loads do not exceed their yield stress, extreme temperatures do not significantly affect the mechanical properties of the multilayered composite. In the final part of the work presented, a complete layered composite with a stiffening rib was tested in a static compression test to experimentally determine the effect of this stiffening rib on the stiffness of the testing panel.

**Figure 24:**
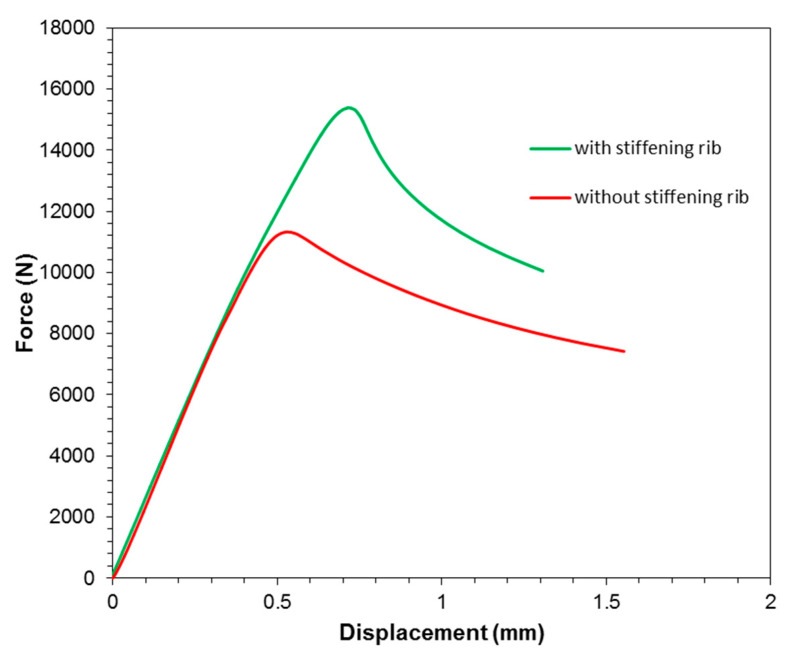
Force–displacement curves obtained from the uniaxial compression test of panels.

Figure 24 summarises the force–displacement curves obtained during compression tests for the variants with and without a stiffening rib. In both cases, the FMLs were fabricated in a 3/2 lay-up configuration. It can be seen that up to a load value of 10,000 N, the courses of both curves are linear and almost identical. The effect of the stiffening rib on the panel stiffness has not been demonstrated here, which suggests that in the adopted configuration, the stiffness is a result of the adhesively bonded GFRP layers and metallic sheets. However, this does not diminish the significance of the stiffening rib, because it has been clearly demonstrated that it has a significant impact on the increase in the critical force at which buckling occurs. For a rib-stiffened panel, the critical force averaged 15,370 ± 110 N, while for the non-stiffened variant, it was 11,430 ± 70 N, which translates into a 34.5% increase in critical force. Such a significant increase in critical force without an increase in the mass of the structure gives broad possibilities regarding the application of the concept presented in the construction of thin-walled structures focused on mass reduction.

**Figure 25:**
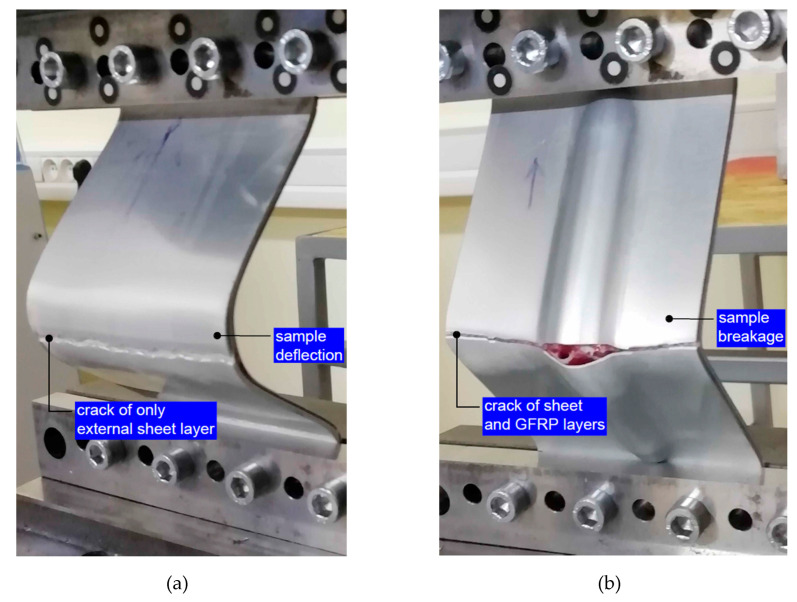
View of the (**a**) FML panel and (**b**) GLARE-based rib-stiffened panel after the compression test.

Based on pictures of the damaged samples (Figure 25), the fracture mechanisms resulting from the geometry of the individual samples can be revealed. Specimens without rib-stiffening were bent after the critical force was exceeded (Figure 25a), as a result of which the outer metallic sheet was broken. In turn, the GLARE-based stiffened samples broke (Figure 25b), which caused the cracking of both the outer sheet metal and the GFRP layer. The fracture occurred on the side of the panel on which the rib appeared. The fracture of the stiffened panel was very rapid, while the cracking of the non-stiffened panel was preceded by the strong bending of the sample.

The aircraft industry uses the technology readiness level (TRL) to define levels of technological maturity. The higher the TRL, the less the development that is needed to prepare a technology for industrial application. Improvements in the aircraft industry are critical to achieving the necessary improvements in almost every aspect of the air transportation system. To improve the performance of aircraft, efforts must mainly be focused on the assurance of sustainable aviation [34]. The most promising among these are drop-in alternative jet fuels that are renewable and lightweight structures. The need to build low-mass structures while maintaining strength and rigidity properties spurs the great interest of aviation manufacturers in new materials, as well as new technologies that provide the opportunity to obtain structures with a high specific strength ratio (strength in relation to mass). The approach presented in the paper to stiffening the GLARE-based thin-walled structure using ISF allowed an increase in the rigidity of the structure. In this paper, a simple demonstration of technology has been presented; however, investigations of a fuselage skin fragment are planned in the future. ISF technology is very flexible and is especially useful when shaping complex spatial rib-shaped stiffeners. In this case, no rigid tools are required. Aluminium alloys are difficult to deform in conditions of conventional sheet metal formation using rigid tools. As ISF is a die-less process, a higher value of sheet deformation may be achieved [35] than that with die forming.

## 4. Conclusions

This manuscript presents the results of the experimental testing of FMLs aimed at the fabrication of a novel solution for a GLARE-based thin-walled structure with a stiffening rib manufactured by incremental sheet formation. Five different methods of composite testing were applied, i.e., a uniaxial tensile test, tensile/shear test, drum peel test, short-beam three-point-bending test and buckling test. The main conclusions drawn are as follows:

- The strength of the 2/1 lay-up FMLs tested at −60 °C and 80 °C varies between 347.9 and 354.7 MPa. However, there is an increase in the stiffness of the specimens stretched at −60 °C compared with those tested at 80 °C. The temperature increase does not weaken the intermolecular forces between the 2024-T3 adherends and the glass/epoxy prepreg, resulting in their similar tensile strength.
- Samples tested at a temperature of 80 °C have a greater degree of elasticity than samples tested at a temperature of −60 °C. The tensile/shear strength of the specimens tested at −60 °C and 80 °C was 19.95 and 19.90 MPa, respectively. The fracture mechanism for the specimens tested at both temperatures analysed is based on the combination of cohesive failure in the resin and adhesive failure in the primer.
- No significant effect of temperature on peel strength was demonstrated in the FML variant with adhesive film. A decrease in the stiffness of the joint was noted at elevated temperature, while a reduction in the peel strength of the adhesion joint is more significant. FMLs with an additional adhesive film exhibited stronger adhesion than FMLs without this film because hardened epoxy resin dominated on the surface of the flexible adherend.
- The load–deflection curves determined through short-beam three-point-bending tests show that after maximum load, each curve displays an irregular fluctuation, demonstrating that multiple failures have taken place. The destruction of the FML layers at the interface with Alclad does not affect the FML’s interlaminar strength in the normal operating conditions of FMLs.
- GLARE-based rib-stiffened panels exhibit a higher critical force in the buckling test than the unstiffened FML panel: a 34.5% increase in critical force is observed.

## Figures and Tables

**Table 1:** Basic mechanical parameters of the 2024-T3 aluminium alloy sheet used.

Thickness *t*, mm	Poisson’s Ratio *ν*	Young’s Modulus *E*, GPa	Yield Stress *R_p0,2_*, MPa	Ultimate Tensile Stress *R_m_*, MPa
0.4	0.33	72.87	302	449
2	0.33	70.75	336	478

**Table 2:** Basic roughness parameters of surfaces of 2024-T3 aluminium alloy sheets.

Roughness Parameter	Sheet Thickness, mm	
	0.4	2
*Sz*, μm	29.4	40.5
*Sp*, μm	13.7	22.9
*Str*	0.32	0.76
*Sv*, μm	15.8	17.6
*Sq*	2.72	4.32
*Sku*	4.53	3.75
*Ssk*	1.18	1.39
*Sa*, μm	1.97	3.36

**Table 3:** Chemical composition of the locations identified in Figure 12, wt%.

EDS Spot	C-K	O-K	Mg-K	Al-K	Cl-K	Cu-K	Mo-L
EDS-1	50.1	32.1	0.4	13.9	1.9	-	1.6
EDS-2	-	-	1.3	93.9	-	4.8	-
EDS-3	-	44.3	0.4	44.7	-	1.3	9.3

**Table 4:** Chemical composition in the locations identified in Figure 15 and Figure 16, wt%.

EDS Spot	C-K	O-K	O-K	Al-K	Mo-L
EDS-1	52.5	8.6	-	9.7	-
EDS-2	-	-	43.2	44.9	11.9
EDS-3	46.3	5.4	33.1	14.1	1.0

**Table 5:** Basic roughness parameters of fractured surfaces of the rigid adherends.

FML Variant	Direction	Roughness Parameter *						
		*Ra*, μm	*Rq*, μm	*Rp*, μm	*Rv*, μm	*Rz*, μm	*Rsk*	*Rku*
with adhesive film	longitudinal	6.06	7.56	12.5	19.5	32.0	−0.37	3.02
	transverse	6.19	6.67	14.1	19.4	33.4	−0.39	2.74
without adhesive film	longitudinal	1.46	1.76	3.87	3.85	7.72	0.10	2.29
	transverse	1.53	1.83	3.67	4.83	8.50	−0.33	2.51

* *Ra*—the average surface roughness, *Rq*—the root mean square deviation of the profile under assessment, *Rp*—the maximum profile peak height, *Rv*—the maximum profile valley depth, *Rz*—the maximum height of the assessed profile, *Rsk*—the skewness, *Rku*—the kurthosis.
